# Géantes lithiases de stase sur vessie iléo-caecale

**DOI:** 10.11604/pamj.2017.28.50.13443

**Published:** 2017-09-20

**Authors:** Hamza Dergamoun, Zayd El Boukili El Makhoukhi

**Affiliations:** 1 Université Mohammed 5, Faculté de Médecine et de Pharmacie de Rabat, Hôpital Ibn Sina, Service d’Urologie A, Maroc

**Keywords:** Exstrophie vésicale, vessie iléo caecal, lithiases, Bladder exstrophy, ileo-caecal bladder, lithiasis

## Abstract

Bladder exstrophy is a malformation characterized by subumbilical anterior abdominal wall and anterior bladder wall deishence. It affects nearly 30.000-40.000 births per year, with a higher prevalence in boys. External continent urinary diversion creates a long-term outcome. However, it is not devoid of complications. We report the case of a 30-year old female patient who had undergone cystectomy with bladder replaced with ileo-caecal segment for bladder exstrophy 13 years earlier. She was followed up for 5 years and then lost to follow up. In March 2017 she presented with painful abdominal heaviness as well as constipation evolving in a context of apyrexy and good general condition. Clinical examination showed good general health, midline laparotomy scar, continent uninvaginated urinary pouch valve. Pelvic examination was unremarkable. Hypogastric palpation showed nonpainful stony-hard mass. Urinary tract without preparation showed calcic opacity 130*110 mm as well as symphysis disjunction. Renal and pelvic ultrasound showed several hyperechogen images with posterior shadow cone occupying the pelvis, two well differentiated kidneys of normal size without ureteric hydronephrosis. CT urography showed 5 big calcium stones with an average density of 730 UH, the most voluminous of which measured 112*101 mm, in the neobladder. Renal function was normal and cytobacteriological examination of urine showed ESBL-producing E coli urinary tract infection sensitive to carbapenems. Treatment was based on suitable antibiotic therapy associated with entero-cystolithotomy involving laborious extraction of 5 big stones. Spectrophotometric examination revealed phospho-ammonium-magnesium stones. The postoperative course was uneventful, post operative urinary tract without preparation showed no stones.

## Image en médecine

L'exstrophie vésicale est une malformation caractérisée par la déhiscence de la paroi abdominale antérieure sous ombilicale et la paroi antérieure de la vessie. Rencontrée environ dans 30000 à 40000 naissance par an, elle semble plus toucher les garçons que les filles, la dérivation externe continente semble donner de bons résultats à long terme elle n'est cependant pas dénuée de complications. Nous rapportons le cas d'une patiente âgée de 30 ans, opérée il y a 13 ans pour extrophie, ayant bénéficie d'une cystectomie avec remplacement par vessie iléo caecal, suivie pendant 5 ans puis perdue de vue. Elle se présente en mars 2017 dans un tableau de douleurs abdominales à type de pesanteur ainsi qu'une constipation évoluant dans un contexte d'apyrexie et de conservation de l'état général. L'examen retrouve une patiente en bon état général avec une cicatrice de laparotomie médiane, la valve de la poche est continente non invaginée avec des touchers pelviens sans particularités. La palpation hypogastrique retrouve une masse dure pierreuse non douloureuse. L'arbre urinaire sans préparation objective une opacité de tonalité calcique 130*110 mm ainsi qu'une disjonction symphysaire. L'échographie rénale et pelvienne objective plusieurs images hyperéchogènes avec cône d'ombre postérieur occupant tous le pelvis, deux reins de tailles normales bien différenciées sans urétèro-hydronéphrose. L'uroscanner retrouve une néo vessie siège de 5 volumineux calculs de densité calcique autour de 730 UH dont la plus volumineuses mesures 112*101mm. La fonction rénale était normale et l'examen cytobactériologique des urines a mis en évidence une infection urinaire à E COLI BLSE sensible aux carbapenèmes. Le traitement a consisté en une antibiothérapie adaptée associée à une enterocystolithotomie avec extraction laborieuse de 5 gros calculs. A l'étude spectrophotométrique les calculs sont de nature phospho-ammoniaco-magnesienne. Les suites opératoires étaient simples, l'arbre urinaire sans préparation post opératoire était stone free.

**Figure 1:**
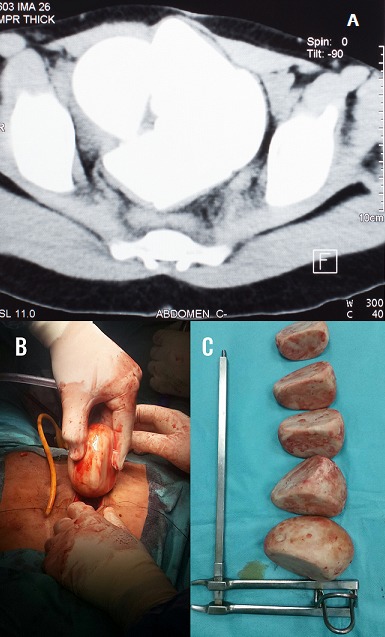
(A) image tomodensitométrique d’une opacité de tonalité calcique siégeant au niveau de la néo vessie; (B) image per-opératoire de l’extraction d’un calculs; (C) image post opératoire de l’ensemble des calculs

